# Assessing the quality of primary care in Haiti

**DOI:** 10.2471/BLT.16.179846

**Published:** 2017-02-08

**Authors:** Anna D Gage, Hannah H Leslie, Asaf Bitton, J Gregory Jerome, Roody Thermidor, Jean Paul Joseph, Margaret E Kruk

**Affiliations:** a Department of Global Health and Population, Harvard T.H. Chan School of Public Health, 665 Huntington Avenue, Boston, MA 02115, United States of America (USA).; b Department of Health Care Policy, Harvard Medical School, Boston, USA.; c Zanmi LaSante, Cange, Haiti.; d Ministry of Health, Port-au-Prince, Haiti.

## Abstract

**Objective:**

To develop a composite measure of primary care quality and apply it to Haiti’s primary care system.

**Methods:**

Using the Primary Health Care Performance Initiative’s framework, we defined four domains of primary care service delivery: (i) accessible care; (ii) effective service delivery; (iii) management and organization; and (iv) primary care functions. We gave each primary care facility in Haiti a quality score for each domain and overall, with poor, fair and good quality indicated by scores of 0.00–0.49, 0.50–0.74 and 0.75–1.00, respectively. We quantified access and effective access to primary care as the proportions of the population within 5 km of any primary care facility and a good facility, respectively.

**Findings:**

Of the 786 primary care facilities in Haiti in 2013, only 332 (43%) facilities were classified as good for accessible care. Fewer facilities were classified as good in the domains of effective service delivery (30; 4%), management and organization (91; 12%) and primary care functions (43; 5%). Although about 91% of the population lived within 5 km of a primary care facility, only an estimated 23% of the entire population – including just 5% of the rural population – had access to primary care of good quality.

**Conclusion:**

Despite an extensive network of health facilities, a minority of Haitians had access to a primary care facility of good quality. Such facilities were especially scarce in rural areas. Similar systematic analyses of the quality of primary care could inform national efforts to strengthen health systems.

## Résumé

**Objectif:**

Élaborer des critères d'évaluation composites de la qualité des soins de santé primaires et les appliquer au système de soins primaires d'Haïti.

**Méthodes:**

En utilisant le cadre de la *Primary Health Care Performance Initiative* (initiative d'amélioration de la performance des soins de santé primaires), nous avons défini quatre aspects de la prestation des services de soins primaires : (i) accessibilité des services; (ii) prestations de services efficaces; (iii) gestion et organisation; et (iv) fonctions de soins primaires. Nous avons attribué à chaque centre de soins primaires d'Haïti un score de qualité (de 0 à 1) pour chacun de ces aspects ainsi qu'un score général. Des scores inférieurs à 0,50, entre 0,50 et 0,74 et supérieurs à 0,75 ont respectivement reflété une qualité mauvaise, satisfaisante et bonne. Nous avons respectivement quantifié les concepts d'accès et d'accès efficace à des soins de santé primaires en tant que pourcentage de la population habitant dans un rayon de 5 km d'un centre de soins primaires et pourcentage de la population habitant dans un rayon de 5 km d'un centre de soins primaires de bonne qualité.

**Résultats:**

Sur les 786 centres de soins primaires recensés en Haïti en 2013, seuls 332 (43%) ont été évalués comme étant de bonne qualité pour le critère «accessibilité des services». Un nombre inférieur de centres ont obtenu un score reflétant une bonne qualité en termes de prestations de services efficaces (30; 4%), de gestion et d'organisation (91; 12%) et de fonctions de soins primaires (43; 5%). Même si à la date de notre étude, près de 91% de la population vivait dans un rayon de 5 km d'un centre de soins primaires, d'après nos estimations, 23% uniquement de la population totale (et même seulement 5% de la population rurale) avait accès à des soins primaires de bonne qualité.

**Conclusion:**

Malgré un réseau étendu de centres de santé, à la date de notre étude, seule une proportion relativement faible d'Haïtiens avaient accès à un centre de soins primaires de bonne qualité. Ces centres étaient particulièrement rares dans les zones rurales. Des analyses systématiques similaires de la qualité des soins primaires pourraient éclairer les efforts nationaux menés pour renforcer les systèmes de santé.

## Resumen

**Objetivo:**

Desarrollar una medida compuesta de la calidad de la atención primaria y aplicarla al sistema de atención primaria de Haití.

**Métodos:**

Utilizando el marco de la Iniciativa de Mejora del Desempeño de la Atención Primaria de la Salud, se definieron cuatro dominios de la prestación de servicios de atención primaria: (i) cuidado accesible; (ii) prestación eficaz de servicios; (iii) gestión y organización; y (iv) funciones de atención primaria. Cada centro de atención primaria de Haití recibió una calificación de calidad para cada dominio y, en general, las calificaciones de calidad escasa, regular y buena se indicaron con calificaciones de 0,00–0,49, 0,50–0,74 y 0,75–1,00 respectivamente. Se cuantificó el acceso y el acceso efectivo a la atención primaria según las proporciones de población en 5 kilómetros de cualquier centro de atención primaria y un buen centro, respectivamente.

**Resultados:**

De los 786 centros de atención primaria en Haití en 2013, únicamente 332 (43%) se clasificaron como buenos para atención accesible. Hubo menos centros clasificados como buenos en los dominios de la prestación eficaz de servicios (30; 4%), gestión y organización (91; 12%) y funciones de atención primaria (43; 5%). A pesar de que el 91% de la población vivía en un radio de 5 kilómetros de un centro de atención primaria, únicamente un 23% estimado de toda la población (incluido el 5% de la población rural) tenía acceso a atención primaria de buena calidad.

**Conclusión:**

A pesar de una extensa red de centros sanitarios, únicamente una minoría de los haitianos tenía acceso a un centro de atención primaria de buena calidad. Dichos centros eran especialmente escasos en zonas rurales. Un análisis sistemático similar de la calidad de la atención primaria podría informar sobre los esfuerzos nacionales para fortalecer los sistemas sanitarios.

## ملخص

**الغرض:**

وضع معيار مركب لجودة الرعاية الأولية وتطبيقه على نظام الرعاية الأولية في هايتي.

**الطريقة:**

قمنا بتعريف أربعة نطاقات تقديم خدمات الرعاية الأولية باستخدام إطار عمل مبادرة أداء الرعاية الصحية الأولية: (1) الرعاية التي يمكن الوصول إليها، و(2) تقديم الخدمات الفعّالة، و(3) الإدارة والتنظيم، و(4) وظائف الرعاية الأولية. نقوم بمنح كل مرفق رعاية أولية في هايتي نقاط جودة لكل نطاق وبصورة شاملة، وتتم الإشارة إلى الجودة السيئة، والمعقولة والجيدة من خلال النقاط 0.00-0.49، و0.50-0.74، و0.75-1.00 على التوالي. كما حددنا مقدار الوصول والوصول الفعال إلى الرعاية الأولية باعتبارها نسب القطاعات السكانية خلال 5 كيلو مترات من أي مرفق رعاية أولية ومرفق جيد، بالتتابع.

**النتائج:**

من 786 مرفق رعاية في هايتي سنة 2013، لم يتم تصنيف إلا 332 (43‏%) مرفقًا كمرفق جيد للرعاية التي يمكن الوصول إليها. وتم تصنيف عدد أقل من المنشآت باعتبارها منشآت جيدة في ضوء نطاقات تقديم الخدمات الفعّالة (30، 4‏%) والتنظيم والإدارة (91، 12‏%)، ووظائف الرعاية الأولية (43، 5‏%). بالرغم من أن حوالي 91‏% من القطاعات السكانية تعيش ضمن 5 كيلو مترات من مرفق الرعاية الأولية، فلا تتوفر سبل الوصول إلى رعاية أولية بجودة نوعية جيدة إلا لما يقدر بنسبة 23‏% من إجمالي السكان، بما في ذلك ‏5‏% من سكان الريف.

**الاستنتاج:**

بالرغم من وجود شبكة واسعة من المرافق الصحية، فإن أقلية من سكان هايتي يمكنهم الوصول إلى مرفق رعاية أولية بجودة جيدة. وكانت المرافق من هذا النوع شحيحة على نحوٍ خاص في المناطق الريفية. ويمكن الاعتماد على التحاليل المنهجية المشابهة لجودة الرعاية الأولية كأساس للمعلومات المقدمة لإفادة الجهود القومية الرامية إلى تعزيز أنظمة الرعاية الصحية.

## 摘要

**目的:**

旨在制定一种综合评估初级医疗质量的方法,并将之应用到海地初级医疗系统中。

**方法:**

我们利用初级卫生保健实施方案 (Primary Health Care Performance Initiative) 的框架,对提供初级医疗服务的四个领域进行了定义: (i) 可及性服务;(ii) 提供有效服务;(iii)管理和组织;以及 (iv) 初级医疗功能。 我们分别从每个领域及整体上对海地的各个初级医疗机构的质量进行了评估,其中 0.00–0.49 表示“质量差”,0.50–0.74 表示“质量中等”,0.75–1.00 表示“质量良好”。 我们分别量化了距离全部初级医疗机构和优质机构 5 公里以内人口中获得及有效获得初级医疗服务者的比例。

**结果:**

在 2013 年,在可及性服务方面,海地的 786 个初级医疗机构中仅有 332(43%) 个被列入“良好”等级。 在提供有效服务 (30; 4%)、管理和组织 (91; 12%) 以及初级医疗功能 (43; 5%) 领域,被列入“良好”等级的机构更少。 尽管约 91% 的人口住在距离初级医疗机构 5 公里范围以内,但是据估计,全部人口中仅有 23% (包括 5% 农村人口)能够获得优质的初级医疗服务。

**结论:**

尽管海地拥有广泛的卫生设施网络,但只有少数海地人能够获得优质的初级医疗服务。 在农村地区,此类设施尤为缺乏。 对初级医疗的质量进行类似的系统化分析有助于海地加强卫生系统建设。

## Резюме

**Цель:**

Разработать обобщенный показатель качества первичной помощи и применить его для системы первичной медико-санитарной помощи на Гаити.

**Методы:**

Используя основные принципы Инициативы по эффективной первичной медико-санитарной помощи (ИЭПМСП), авторы определили четыре аспекта оказания услуг первичной медико-санитарной помощи: 1) доступность медицинской помощи; 2) эффективность оказания услуг; 3) управление и организация; 4) функции первичной медико-санитарной помощи. Авторы оценили качество каждого учреждения первичной медико-санитарной помощи на Гаити по каждому аспекту и в целом в баллах: результат от 0,00 до 0,49 балла означал плохое качество, результат от 0,50 до 0,74 балла — среднее, результат от 0,75 до 1,00 балла — хорошее. Доступность и эффективная доступность первичной медико-санитарной помощи оценивались количественно как доля населения, проживающая в радиусе 5 км от любого учреждения первичной медико-санитарной помощи и от учреждения, качество которого, согласно оценке, было хорошим, соответственно.

**Результаты:**

В 2013 году из 786 учреждений первичной медико-санитарной помощи на Гаити только 332 (43%) были отнесены к хорошим с точки зрения доступности медицинской помощи. Меньшее количество учреждений было отнесено к хорошим с точки зрения эффективности оказания услуг (30; 4%), управления и организации (91; 12%) и функций первичной медико-санитарной помощи (43; 5%). Хотя около 91% населения проживало в радиусе 5 км от учреждений первичной медико-санитарной помощи, согласно подсчетам, только 23% от всего населения, включающие лишь 5% сельского населения, имели доступ к первичной медико-санитарной помощи хорошего качества.

**Вывод:**

Несмотря на развитую сеть учреждений здравоохранения, меньшая часть гаитян имела доступ к учреждениям первичной медико-санитарной помощи хорошего качества. Особый недостаток таких учреждений наблюдался в сельской местности. С помощью сходных систематических анализов качества первичной медико-санитарной помощи можно было бы получить информацию для проведения мероприятий по укреплению систем здравоохранения на национальном уровне.

## Introduction

Thirty years after the Declaration of Alma-Ata, the 2008 *World Health Report* declared that primary health care was a global priority “now more than ever”.^1^ Primary care forms the cornerstone of a functional health system. High-quality primary care systems can improve health outcomes, increase equity in health care and optimize efficient use of resources.^2^^–^^4^ In low- and middle-income countries, however, primary care is often poor, with a general lack of provider effort, high rates of misdiagnosis and incorrect treatment, and long wait times.^5^^–^^8^

Research on the quality of primary care includes investigations of provider behaviour and knowledge,^5^ programme evaluations^9^ and small-scale case studies.^10^ Broader assessments of primary care systems, particularly in the wake of conflict^11^ or natural disaster,^12^ have included the development of balanced scorecards. These scorecards have focused on infrastructure inputs and community perspectives and given relatively little attention to the processes of care. One limitation of the research in this field is the lack of a comprehensive definition of primary care quality that is applicable across contexts and countries.

In an effort to guide quality measurement and improvement in the field of primary care, the Primary Health Care Performance Initiative reviewed over 40 different conceptual frameworks of primary care and consolidated them into a single framework.^13^ This framework, which is still evolving, unifies previous work into five key areas: system, inputs, service delivery, outputs and outcomes. An important contribution of this framework is the delineation of the service delivery area, a critical but understudied element of primary care quality, into five interconnected domains. These are population health management, e.g. community engagement; facility management and organization; access to care that is timely and affordable; the availability of effective services; and high-quality primary health care. The final domain follows from the others and encompasses Starfield’s formulation of primary care’s roles and functions: coordination, comprehensiveness, continuity and first-contact access.^14^

The development of new metrics based on this framework is a critical next step in assessing the quality of the delivery of primary health care. Metrics that align with updated theoretical frameworks and shed light on the quality of care provided to patients are needed to understand primary care performance more fully. Such metrics can help health ministries identify shortfalls in the provision of quality primary care and prioritize appropriate action.

Given its poor population health outcomes and its recent attempts to build a strong primary care system, Haiti presents a compelling case study of primary care quality. Life expectancy at birth is 65 years, and mortality among children younger than five years is more than double that in the neighbouring Dominican Republic.^15^ There is only one doctor or nurse per 3000 population and public sector health spending is among the lowest in the world. An earthquake in January 2010 placed further strain on the health system and caused tremendous loss of life and immense physical damage, destroying 50 health facilities.^16^^,^^17^ Despite natural disasters, poverty and underinvestment in health, Haiti has achieved some notable health gains in recent decades, including a steady decline in mortality among children younger than five years.^15^

In 2008, Haiti’s primary care system was classified as selective, with targeted application of high-impact interventions in facilities that, in general, struggled with the provision of routine care.^18^ In 2007, the Haitian Ministry of Health’s National Quality Committee launched HIVQual, a system for data collection, based on electronic medical records, designed to measure and improve the quality of services for people living with the human immunodeficiency virus (HIV).^19^ In 2012, this system was expanded to cover some non-HIV services’ care and to reach a larger number of facilities.^19^ As global health policy pivots towards universal health coverage and to tackling the broad array of health challenges outlined in the sustainable development goals,^20^^,^^21^ it is an opportune moment to test a methodology for assessing coverage of comprehensive, high-quality primary care.

Below, we describe the development of a theoretically grounded metric of primary care quality, based on existing survey and geospatial data, and the metric’s application in measuring the quality of Haiti’s primary care system. We drew on a census of Haiti’s health facilities to evaluate the performance of the country’s primary care system in 2013, describe geographical access to quality care and assess the disparities in such access. To highlight the challenges and opportunities of measurement in this understudied area, we focused on the service delivery component of the Primary Health Care Performance Initiative’s framework.

## Methods

### Study sample

We used data from the Service Provision Assessment, which is a census survey of health facilities conducted in Haiti in 2013 by the Demographic and Health Survey Program. The census included a facility assessment, a questionnaire for health-care providers, observations of sick child, antenatal care and family planning visits, and exit interviews with observed clients. We limited our analysis to the data collected on outpatient primary care facilities, i.e. dispensaries and health centres with or without beds.^22^

We also used WorldPop maps to obtain estimates of the 2015 population density of Haiti, at a resolution of 100 m^2^.^23^

### Measuring primary care quality

**Fig. 1:**
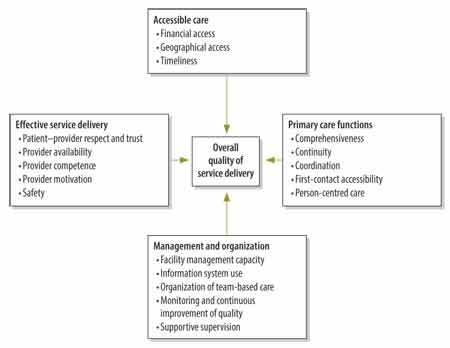
**Conceptual framework of quality in primary health care**

Source: Adapted from the Primary Health Care Performance Initiative’s framework,^13^ for use in Haiti.

We developed metrics of service delivery quality following the Primary Health Care Performance Initiative’s framework. Several modifications were required to adapt the framework for health facility assessment (Fig. 1). We excluded the domain “population health management”, because of a lack of relevant facility-related data. For clarity, we also altered the labels for two of the domains, using “effective service delivery” for the availability of effective services and “primary care functions” for high-quality primary health care.^14^

We reviewed the data available in the survey and selected 28 indicators that most appropriately matched each of the quality subdomains included in our analysis. For this selection, we were guided by the Primary Health Care Performance Initiative’s method note.^13^ Each indicator is a proportion or an index that ranges from 0 to 1. For example, the indicator “sick child did not first visit traditional healer” measures first-contact access to a facility as the proportion of sick children who came to the facility for care without first visiting a traditional healer. All selected indicator definitions are available from the corresponding author. Within the survey data, we were unable to find relevant indicators for two of the subdomains that we wished to investigate: geographical access and the organization of team-based care. As people need to be able to access health facilities to benefit from quality care, we used the WorldPop maps to determine geographical access to facilities.

For each primary care facility, we calculated a score for each of four service delivery domains: (i) accessible care; (ii) effective service delivery; (iii) management and organization; and (iv) primary care functions. Each of these scores, which could range from 0 to 1, was the mean of all the indicators under the domain. As we considered the four domains to be equally important elements of quality primary care, we took the mean of the four scores calculated for each facility as the overall measurement of the quality of the facility’s service delivery for primary care.

Although the census covered all but two of the health facilities in Haiti in 2013, two of the survey tools, i.e. clinical observations and patient interviews, were applied only in a selected subset of facilities. For each indicator included in our analysis, we used multiple imputation to generate five versions of a completed data set for all quality indicators. We based the imputation on observed covariates, e.g. management type and urban, and the non-missing indicators.

Finally, we assessed the distribution of indicators across facilities and sought valid groupings of better and worse quality. Given the lack of universally defined minimum quality thresholds and the rudimentary nature of many of the indicators included in our analysis, we divided the facility scores into three categories of quality. Scores of less than 0.50, 0.50–0.74 and at least 0.75 were considered indicative of poor, fair and good quality, respectively.

### Covariates

We defined each 100 m^2^ block of population as an urban or rural population using the census’ urban or rural classification of the facility nearest to the centre of the block. As a sensitivity check, we also defined an urban population as one in which there were at least five people per 100 m^2^ block.

### Analysis

We calculated descriptive statistics of the primary care facilities with non-response weights. We summarized mean values and uncertainty intervals for each indicator, domain and overall quality score for service delivery. As the data we analysed provided a census of the primary care facilities in Haiti in 2013, the uncertainty intervals that we calculated indicate the measurement error attributable to missing data.^24^ Using inverse distance-weighted interpolation, we mapped, across Haiti, the quality of the primary care available to a nearby population. In the resultant map, the colour of each 100 m^2^ block indicates whether the quality of the nearest primary care facility was poor, fair or good. We used the global Moran’s *I* statistic, which tests for the presence of spatial autocorrelation,^25^ to investigate whether facilities of good or poor quality, in terms of each of the four domains of interest, were clustered geographically. Moran’s *I* can range from −1 to 1. In our analyses, positive *I* values would indicate that primary care facilities of similar quality were clustered together. We defined proximity using an inverse-distance weight matrix.^26^ In keeping with prior research on physical access to care in Haiti,^27^ we calculated the percentages of the entire Haitian population, rural population and urban population living within 5 km of any facility and within the same distance of a facility with a good overall score. Finally, we mapped the areas that lay within 5 km of any facility and a facility with a good overall care score.

Multiple imputation was conducted in R 3.2 (R Core Team, Vienna, Austria). All other analyses were conducted in Stata version 14.0 (StataCorp, LP, College Station, United States of America). We used QGIS version 2.12^28^ to map the data.

### Ethical approval

The Harvard University Human Research Protection Program categorized this secondary analysis of data as exempt from human subjects review.

## Results

**Fig. 2:**
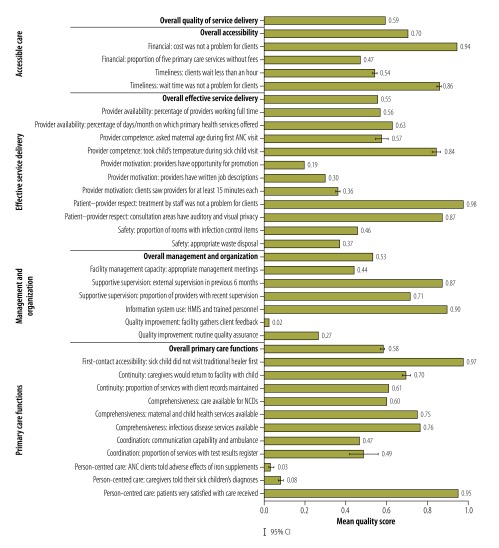
Indicators of the quality of the delivery of primary care services, Haiti, 2013

ANC: antenatal care; CI: confidence interval; HMIS: health management information system: NCDs: noncommunicable diseases.

Note: Error bars illustrate the between-imputation variance in the proportions. Error bars not visible when variance <1%.

The survey obtained detailed data from 905 (99.8%) of the 907 health facilities in Haiti in 2013, 786 of which were primary care facilities and included in the analysis (Table 1). Most primary care facilities were classified as rural, although there was a high concentration of primary care facilities in and around Port-au-Prince. Fig. 2 summarizes the performance of the primary care facilities across the four domains of primary care service delivery. At the average facility, 86% and 94% of clients, respectively, stated that they did not find wait times or the costs of care to be a problem, even though about half of all primary care services required payment and over half of the primary care facilities had mean wait times in excess of one hour. Large gaps in quality were evident in the metrics for the availability of effective services. The indicators for provider motivation and safety were found to be especially low. Basic elements of clinical care were not universally followed. For example, at the average facility only 57% of the providers asked about maternal age at a first visit for antenatal care. Low quality scores for primary care functions were partially attributable to poor provider communication. Under management and organization, only 2% (18) of the primary care facilities had a system for gathering feedback from their clients and nearly three-quarters (577) did not have routine quality assurance processes. For their overall quality of service delivery, the primary care facilities in Haiti achieved a mean score of 0.59.

**Table 1:** Characteristics of primary care facilities, Haiti, 2013

Characteristic	No. of facilities^a^ (%) *n* = 786
**Setting**	
Rural	526 (67)
Urban	260 (33)
**Facility type**	
Health centre with beds	129 (16)
Health centre without beds	297 (38)
Dispensary	358 (46)
**Management type**	
Public	292 (37)
Private, not for profit	142 (18)
Private, for profit	180 (23)
Faith-based	170 (22)
**With inpatient or maternity beds**	355 (46)
**Offers pharmacy services**	766 (98)
**Offers laboratory services**	544 (69)

^a^ Each facility was staffed by a mean of 1.15 (standard deviation, SD: 3.26) generalist doctors, generalist surgeons and/or specialist doctors and a mean of 3.87 (SD: 4.71) auxiliary nurses, midwives, nurse/midwives and/or nurses.

**Fig. 3:**
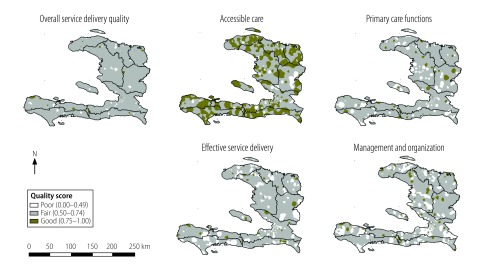
Population coverage of primary care of poor, fair or good quality, Haiti, 2013

Note: The maps show the populations for which the nearest primary care facility provided care that was scored as poor, fair or good by domain and overall. The administrative boundaries shown are based on data from the GADM spatial database.^29^

Source: Authors, using data from the GADM spatial database and Service Provision Assessment.^29^

Most facilities (84%; 660/786) had fair overall quality of care and only 15 had good overall quality (Fig. 3). Nearly half of the 786 primary care facilities (43%; 332) offered good accessible care but only 4% (30) and 6% (42) ranked as good in terms of effective service delivery and primary care functions, respectively. Tests of spatial autocorrelation provided evidence of geographical clustering in terms of overall quality and the quality of accessible care, effective service delivery and management and organization. However, the magnitude of every such association was small (available from the corresponding author), indicating that considerable geographical heterogeneity in the quality of primary health care existed.

**Fig. 4:**
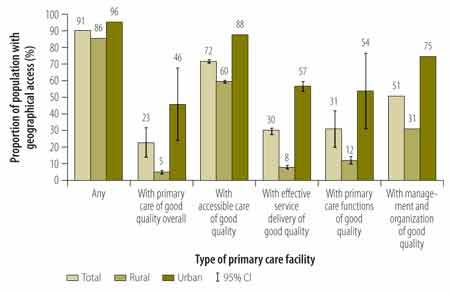
**Geographical access to any primary care facility and facilities providing primary care of good quality, Haiti, 2013**

**Fig. 5:**
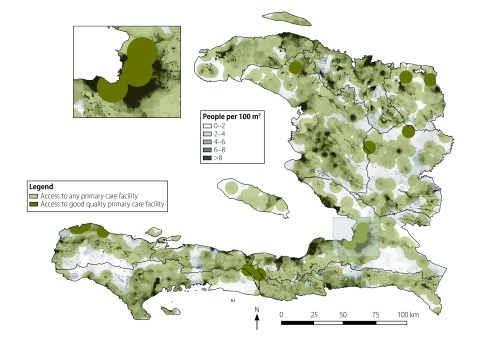
Map of the geographical access to any primary care facility and facilities providing primary care of good quality, Haiti, 2013

Note: A person was considered to have geographical access to a facility if they lived no more than 5 km, measured linearly, from that facility. The magnified box presents the capital, Port-au-Prince. The administrative boundaries shown are based on data from the GADM spatial database.^29^

Source: Authors, using data from the GADM spatial database, Service Provision Assessment survey and WorldPop population density.^29^

Fig. 4 illustrates geographical access of Haiti’s entire, rural and urban populations to primary care. At the time of the census, an estimated 90.6% of Haiti’s population lived within 5 km of a primary care facility. Although almost 8 million people, that is, 72% of the national population, lived within 5 km of a primary care facility providing good accessible care, smaller numbers lived as close to facilities providing good management and organization (51%), good primary care functions (31%) or good effective service delivery (30%). Compared with rural dwellers, urban residents had higher access to good quality care along all of the domains. For example, only 8% of rural dwellers but 57% of urban residents had access to effective service delivery of good quality. Similar trends were observed when, in a sensitivity check, we defined an urban population as one in which there were at least five people per 100 m^2^ block (available from the corresponding author). At the time of the census, only an estimated 2.5 million people (of 10.65 million total) in Haiti lived within 5 km of a facility with good overall quality of care (Fig. 5).

CI: confidence interval.

Note: A person was considered to have geographical access to a facility if they lived no more than 5 km, measured linearly, from the facility. Error bars illustrate the between-imputation variance in the proportions. Error bars not visible when variance <1%.

## Discussion

This study applies a novel approach to measuring the quality of primary care services in Haiti in terms of four quality domains and using existing data sources. We found that the Haitian population’s access to primary care of good quality in 2013 was very limited: while 91% of the population lived within 5 km of a primary care facility, only 23% lived within 5 km of a facility with service delivery of good quality. The mean overall score for the quality of service delivery was only 0.59, indicating there are many gaps in the provision of high quality primary care. In general, the primary care facilities performed reasonably well on access indicators but poorly in terms of effective service delivery and management and organization, with particular deficits in provider motivation and quality improvement. As the quality indicators based on clients’ responses tended to be more positive than those that had been more objectively assessed, it appears that clients may have had low expectations when seeking care. We found limited evidence of geographical clustering of quality. The quality of service delivery varied substantially from facility to facility within both rural and urban areas, although it was, in general, relatively poor in rural areas.

Most previous studies of the quality of primary care in Haiti have used service utilization, for example, by the numbers of antenatal care visits or vaccination rates, as an indicator of quality.^30^^,^^31^ Our study, which incorporated indicators for preventive services and curative services for communicable and noncommunicable diseases, moved beyond utilization to consider the service environment and the whole process of care during a primary care visit. Like a recent review of the literature on the service delivery experience of patients using primary care,^5^ our study indicates profound gaps in the provision and receipt of primary care of good quality. In Haiti, as elsewhere, robust quality measurement is a crucial input to the ongoing efforts to improve the quality of primary care. A recent assessment of the impacts of a Haitian programme to improve HIV services found that the programme had triggered a broad and beneficial change in the culture of quality improvement.^32^

Our application of the Primary Health Care Performance Initiative’s framework for the delivery of primary care services yielded several insights. The framework currently represents the most comprehensive and well-defined approach to primary care quality – with a particular emphasis on the often undermeasured area of service delivery. However, we feel that the framework’s current domain labels could be made more intuitive and, more substantively, that the framework leaves patient safety as a poorly defined construct in the context of primary care delivery. There is also a lack of comprehensive data covering all of the relevant domains and subdomains. We identified several priorities for improving the measurement of primary care quality (Box 1). The framework, the tools used in surveys of health facilities and the links between the results of facility surveys and national policy processes all need critical review. Additionally, while the measurement of primary care quality is intrinsically important, future work should also link the quality of care to population health outcomes.

Box 1 **Priorities in improving the measurement of primary care quality**

- Better definitions, e.g. in patient safety;
- Better data, e.g. in first-contact accessibility, continuous care, coordinated care, population health management and the team-based management of care;
- More efficient measures, e.g. identification of the smallest set of indicators needed to measure quality effectively and definition of the roles of routine information systems and special studies in providing the data required to monitor the quality of primary care;
- More meaningful measures, e.g. determination of appropriate minimum thresholds for good quality and the best methods to compare such thresholds across different settings;
- Investigation of the population served by a primary care facility, particularly the difference between planned catchments and actual utilization and the identification of the data needed, from the population, to conduct equity analyses on the quality of care;
- Investigation of the best ways to translate the results of quality measurement into effective methods of quality improvement.

Our study had several other limitations. Although we performed multiple imputation to account for missing data, such data could still have introduced bias into the analysis – i.e. if the facilities without observations and patient interviews differed systematically, in ways not captured by the covariates used in imputation, from the other facilities. In addition, our use of linear distance to estimate geographical access may have been misleading. Especially in rural and mountainous regions, the distance that an individual has to travel between two points may be much greater than the linear distance between those points. Detailed data on road networks and quality and on transportation costs would strengthen our observations. Finally, as Service Provision Assessment data are only available for a small set of countries, the approach that we followed may not be applicable in many other settings, although indicators similar to the ones that we investigated are available from other health facility assessments.^33^^,^^34^

Our results have several implications for primary health care in Haiti. As an immediate next step, they can be used, by funders, planners, policy-makers and practitioners, to compare performance within administrative areas and to identify the best- and worst-performing facilities within each area. This should allow improvement interventions to be better targeted at particular facilities and at known weaknesses. Despite Haiti’s challenging topography, primary care of good quality has been achieved, and should be more widely achievable, in all areas. Most primary care facilities of poor quality in Haiti are close to, and could learn from, a facility of good quality. Elements of service delivery quality that were found to be absent from almost all of Haiti’s primary care facilities, e.g. the gathering of client feedback and good provider communication, should be targeted for improvement and regular measurement. Strategies to address these gaps could include provider training on patient-centred approaches,^35^ the strengthening of data feedback loops for providers^36^ and the enhancement of managerial supervision.^37^

More broadly, our study complements the efforts of Haiti’s National Quality Committee over the past decade to monitor and improve the quality of health services, particularly via the development of broader health services based on HIV care.^32^ The comprehensive definition of quality that we employed provides an opportunity to spur the development of standardized systems to monitor and improve the quality of primary care services and to complement Service Provision Assessments or similar periodic evaluations. The Haitian Ministry of Health could build on its experience with programmes to improve the quality of HIV services to adapt and apply strategies, systems and tools for the routine monitoring of indicators across all dimensions of primary care quality. Work is already underway to compare the utilization and patient-outcome indicators from other quality measurement approaches, such as HIVQual,^19^ with the indicators that we investigated. Only with the routine measurement of quality and evidence-based quality improvement can the quality of services offered at primary care level improve health outcomes and meet the legitimate expectations of Haitians.
